# A Novel Viral SOCS from Infectious Spleen and Kidney Necrosis Virus: Interacts with Jak1 and Inhibits IFN-α Induced Stat1/3 Activation

**DOI:** 10.1371/journal.pone.0041092

**Published:** 2012-07-23

**Authors:** Chang-Jun Guo, Li-Shi Yang, Ying-Fen Zhang, Yan-Yan Wu, Shao-Ping Weng, Xiao-Qiang Yu, Jian-Guo He

**Affiliations:** 1 MOE Key Laboratory of Aquatic Product Safety/State Key Laboratory for Biocontrol, School of Marine Science, Sun Yat-sen University, Guangzhou, PR China; 2 State Key Laboratory for Biocontrol, School of Life Sciences, Sun Yat-sen University, Guangzhou, PR China; 3 Division of Cell Biology and Biophysics, School of Biological Sciences, University of Missouri-Kansas City, Kansas City, Missouri, United States of America; Johns Hopkins School of Medicine, United States of America

## Abstract

Interferon (IFN)-induced Janus kinase (Jak)/signal transducer and activator of transcription (Stat) pathway is important in controlling immune responses and is negatively response-regulated by the suppressor of cytokine signaling (SOCS) proteins. However, several viruses have developed various strategies to inhibit this pathway to circumvent the anti-viral immunity of the host. The infectious spleen and kidney necrosis virus (ISKNV) is the type species of the genus *Megalocytivirus* in the family *Iridoviridae* and a causative agent of epizootics in fish. ISKNV ORF103R encodes a predicted viral SOCS (vSOCS) with high homology to the vertebrate SOCS1, but lacks a SOCS-box domain. Interestingly, vSOCS only exists in the genus *Megalocytivirus*. ISKNV-vSOCS can block the IFN-α-induced Jak/Stat pathway in HepG2 cells. Over-expression of ISKNV-vSOCS inhibited the activities of IFN-stimulated response element (ISRE) promoter; however, the inhibitions by ISKNV-vSOCS were dose-dependent. ISKNV-vSOCS interacted with Jak1 protein and inhibited its tyrosine kinase activity in vitro. ISKNV-vSOCS also impaired the phosphorylation of Stat1 and Stat3 proteins and suppressed their activations. The point mutations (F18D, S66A, S85A, and R64K) of ISKNV-vSOCS significantly impaired the inhibition of IFN-α-induced ISRE-promoter activation. In conclusion, vSOCS inhibits IFN-α-induced Stat1/Stat3 signaling, suggesting that Megalocytivirus has developed a novel strategy to evade IFN anti-viral immunity via vSOCS protein.

## Introduction

Janus kinase (Jak)/signal transducer and activator of transcription (Stat) signal transduction pathway is important controlling immune responses [1]. The Jak/Stat pathway is induced by a large number of cytokines, growth factors, and hormones, such as interferon (IFN)-α and IFN-γ, interleukin (IL)-1, IL-2, IL-4, IL-5, IL-6, and IL-10, insulin, and growth hormone [2], [3], [4]. The binding of cytokines to their cell-surface receptors induces the dimerization of the receptors, which activates the associated Jaks to phosphorylate the receptors [5]. Specific Stats are recrutied by the receptors and are phosphorylated by the activated Jaks [6]. The phosphorylated Stats then dissociate from the receptors and form an activated dimer or tetramer that translocates into the cell nucleus and binds to a specific DNA element to induce the transcription of target genes [7].

Suppressor of cytokine signaling (SOCS) proteins constitute a class of inducible negative feedback regulators of the Jak/Stat signaling transduction pathway [8]. The SOCS family in mammals consists of eight members: SOCS1–SOCS7 and cytokine-inducible SH2-containing protein (CIS) [9]. Two novel members of the SOCS protein family, namely, SOCS8 and SOCS9 have been recently identified in fish [10]. SOCS family members share a similar architecture, including a variable N-terminal region, an extended SH2 sub-domain (ESS), a central SH2 domain, and a C-terminal SOCS box [11]. SOCS1 and SOCS3 contain an additional kinase inhibitory region (KIR) that is essential for the inhibition of kinase activity [12]. SOCS family members share multiple complementary mechanisms that negatively regulate cytokine signaling [13]. First, SOCS1 and SOCS3 proteins directly bind to Jaks through the SH2 domain and inhibit Jak activity [14], [15]. Second, SOCS proteins compete with each other on the acquisition of Stat proteins or bind to different phosphorylated tyrosine residues of cytokine receptors [16]. Third, SOCS proteins obtain the ubiquitin proteasome system to degrade Jaks or other signaling molecules via the SOCS box [17].

IFN are important cytokines for innate immunity and for host protection from viral infections through the Jak/Stat signaling transduction pathway [18], [19]. Several viruses have developed various strategies to circumvent the IFN-induced anti-viral immunity of the host [20]. However, it has not been reported that viruses can use viral SOCS (vSOCS) proteins to inhibit the Jak/Stat signaling pathway.

Infectious spleen and kidney necrosis virus (ISKNV) was identified and isolated from mandarin fish (*Siniperca chuatsi*) in 1998 [21]. It is an icosahedral cytoplasmic dsDNA virus belonging to the family *Iridoviridae*. The iridovirus has a broad host range, which includes insects, fish, amphibians, and reptiles. Most members of the iridovirus family have been associated with serious diseases in frogs and fishes [22]. According to the eighth report of the International Committee on Taxonomy of Viruses, the family *Iridoviridae* has been subdivided into five genera, including *Iridovirus, Chloriridovirus, Ranavirus, Lymphocystivirus,* and *Megalocytivirus* [23]. As a species in the genus *Megalocytivirus*, ISKNV infects mandarin fish (*Siniperca chuatsi*), zebrafish (*Danio rerio*), tetraodon (*Tetraodon nigroviridis*), largemouth bass (*Micropterus salmoides*), and more than 50 species of marine fish [24]. Megalocytiviruses have attracted attention because they are highly infectious and pathogenic to fish, causing severe economic losses in Asian aquaculture [25]. The ISKNV genome has been previously sequenced and analyzed in our laboratory [26]. The genome contains 124 potential open reading frames (ORF), with coding capacities ranging from 40 to 1208 amino acids. ISKNV ORF103R was found to encode a predicted protein of 133 amino acids and contain an SH2 domain.

The present study analyzed the characteristics of ISKNV ORF103R and demonstrated that this ORF encoded a novel vSOCS protein which inhibited IFN-α-induced Jak/Stat signal transduction pathway. Interestingly, vSOCS proteins only exist in the genus *Megalocytivirus* of the *Iridoviridae* family. This study was the first to report a vSOCS protein that could impair the Stat1/Stat3 signaling pathway.

## Results

### Computer-assisted Analysis of Sequences and Bioinformatics

PCR was performed using ISKNV genomic DNA and cDNA extracted from the spleens of ISKNV-infected mandarin fish as templates to confirm the presence of ISKNV ORF103R. cDNA fragments of ∼400 bp were obtained. The sequencing results showed that the DNA sequences of two fragments were identical and perfectly matched ISKNV ORF103R. ISKNV ORF103R had 402 bp and encoded a predicted protein of 133 amino acids. SMART analysis and BLASTp results showed that ISKNV ORF103R encoded a protein having a conserved SH2 domain and was highly homologous to vertebrate SOCS1 proteins. Thus, this protein encoded by ISKNV ORF103R was named as ISKNV-vSOCS. Interestingly, similar *vsocs* genes were found in all members of the genus *Megalocytivirus* with genome sequences, including orange-spotted grouper iridovirus (OSGIV), rock bream iridovirus (RBIV), and large yellow croaker iridovirus (LYCIV) [27], [28], [29]. In addition, *vsocs* genes only exist in the genus *Megalocytivirus* of the *Iridoviridae* family and not in any other virus species/stains.

**Figure 1:**
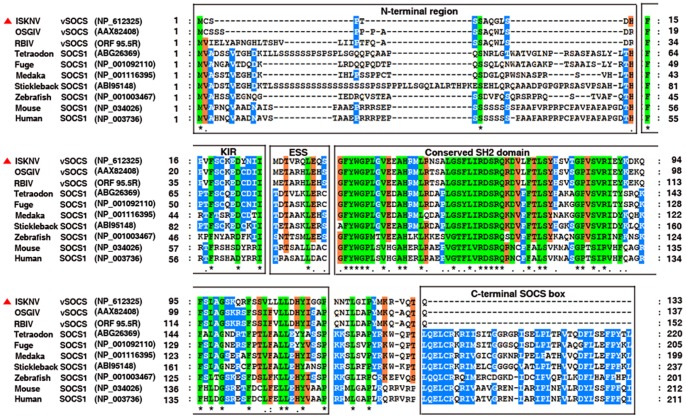
Multiple sequence alignments of megalocytiviruses vSOCS proteins with vertebrate SOCS1 proteins. The amino acid sequences of vSOCS proteins from megalocytiviruses (ISKNV, RBIV, OSGIV) and SOCS1 proteins from stickleback (*Gasterosteus aculeatus*), medaka (*Oryzias latipes*), tetraodon (*Tetraodon nigroviridis*), fuge (*Takifugu rubripes*), zebrafish (*Danio rerio*), mouse (*Mus musculus*), and human (*Homo sapiens*) were analyzed using ClusterX v1.83. About 100%, 80%–100%, and 60%–80% identities are indicated in green, blue, and red, respectively. Identical (*) and similar (· or :) residues are also indicated below the alignment. Predicted domains are indicated by boxes.

vSOCS proteins (from ISKNV, RBIV, and OSGIV) and SOCS1 proteins from different organisms, such as human, mouse, zebrafish, tetraodon, stickleback, fuge, and medaka were then compared (Figure 1). vSOCS proteins shared similar architecture with vertebrate SOCS1 proteins, including a variable N-terminal region, KIR/ESS, and a conserved SH2 domain, but lacked a C-terminal SOCS box. ISKNV-vSOCS is most similar (79% identity) with vSOCS proteins from OSGIV (ORF 99R) and RBIV (ORF 95.5, from nucleotides 91477 to 91935 in the genomic DNA) and with two members of the genus *Megalocytivirus*. Moreover, ISKNV-vSOCS showed higher similarity with SOCS1 proteins from fishes than those from mammals, with 65%, 62%, 61%, 60%, and 51% identities to SOCS1 of stickleback (*Gasterosteus aculeatus*), medaka (*Oryzias latipes*), tetraodon (*Tetraodon nigroviridis*), fuge (*Takifugu rubripes*), and zebra fish (*Danio rerio*), whereas 42% and 41% identities to SOCS1 of mouse and human, respectively.

**Figure 2:**
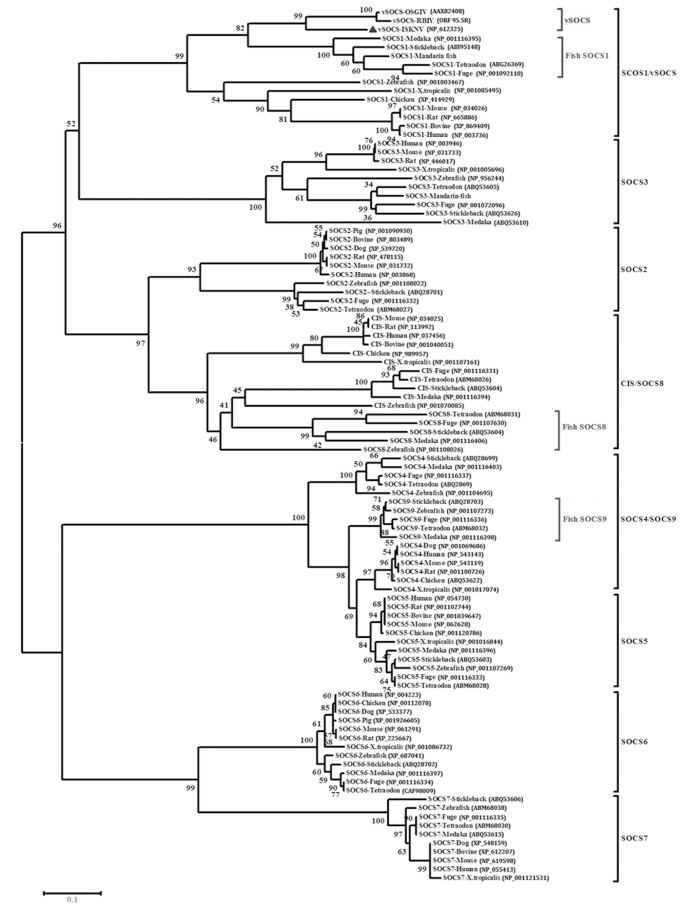
Phylogenetic analysis of vSOCS and SOCS family proteins from various species. The phylogenetic tree was constructed by bootstrap N-J method using MEGA 4.0. Bootstrap value was indicated at the node. Homologous vSOCS protein sequences from the three sequenced members of the genus *Megalocytivirus* (ISKNV, OSGIV, RBIV), mandarin fish (*Siniperca chuatsi*), stickleback (*Gasterosteus aculeatus*), medaka (*Oryzias latipes*), tetraodon (*Tetraodon nigroviridis*), fuge (*Takifugu rubripes*), zebrafish (*Danio rerio*), chicken (*Gallus gallu*), x.tropicalis (*Xenopus tropicalis*), bovine (*Bos taurus*), pig (*Sus scrofa*), dog (*Canis familiaris*), mouse (*Mus musculus*), rat (*Rattus norvegicus*), and human (*Homo sapiens*) were retrieved from the GenBank or the Ensemble database (the accession numbers indicated) and analyzed.

Protein sequences of SOCS family members were sought and a phylogenetic analysis was performed to further investigate the possible evolutionary origin of vSOCS (Figure 2). The phylogenetic results showed that the vSOCS proteins of ISKNV, OSGIV, and RBIV formed a monophyletic group (100% bootstrap support), which, by evolutionary analysis, was more closely related to the monophyletic group of fish SOCS1 proteins than other vertebrate SOCS1 proteins. These two monophyletic groups and vertebrate SOCS1 proteins comprised a larger monophyletic group, the vSOCS/SOCS1 subfamily, which differed from the SOCS2, SOCS3, SOCS4/fish SOCS9, SOCS5, SOCS6, SOCS7, and CIS/fish SOCS8 subfamilies (Figure 2).

### ISKNV-vSOCS Inhibits the Interferon-α-mediated Reporter Gene Activities

**Figure 3:**
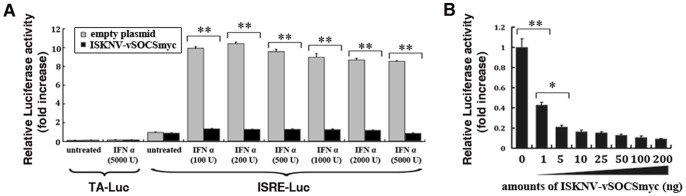
Activities of the ISRE-promoter luciferase reporter genes. (A) IFN-α-responsive ISRE-luc promoter activity. The cells were treated with recombinant IFN-α (100∼5000 U) for 8 h at 24 h after transfection. The gray columns represent the RLA levels in cells transfected with empty plasmid, whereas the black columns represent the RLA levels in cells transfected with ISKNV-vSOCSmyc plasmid. RLA level in cells transfected with TA-luc reporter gene instead of the ISRE-luc reporter gene was used as a negative control. RLA levels of cells transfected with empty plasmid without stimulation were arbitrarily set as 1. (B) Activities of reporter genes in cells transfected with increasing amounts of ISKNV-vSOCSmyc plasmid. Cells were transfected with different amounts of ISKNV-vSOCSmyc plasmid (1∼200 ng), treated with IFN-α (5000 U) for 8 h, and then ISRE-luc activity was analyzed. RLA levels in cells transfected with empty plasmid after IFN-α treatment were arbitrarily set as 1. Error bars represent the mean ± S.D. (n = 3). **P<0.01.

The activities of IFN-stimulated response element (ISRE)-promoter (ISRE-luc) reporter genes in response to IFN-α were observed after transfection with ISKNV-vSOCSmyc plasmid to investigate the function of ISKNV-vSOCS. Firefly luciferase activity was normalized to Renilla luciferase activity. The relative luciferase activity (RLA) levels of the cells transfected with empty plasmid without stimulation (control sample) were arbitrarily set as 1. Other RLA levels were presented as fold-increases over that of the control sample. As negative controls, the cells were transfected with a control reporter gene (TA-luc). The RLA levels were very low (<0.4) with or without IFN-α stimulation (Figure 3A, bar groups 1 and 2). Without IFN-α stimulation, the RLA levels were also low (∼1.0) in cells transfected with empty plasmid and ISKNV-vSOCSmyc plasmid (Figure 3A, bar group 3). The RLA levels in the cells transfected with the control plasmid significantly increased (8.5–10.4 folds) after IFN-α (100 U to 5000 U) stimulation (Figure 3A). However, RLA levels in cells transfected with ISKNV-vSOCSmyc remained at a low level (1.1–1.3 folds) (Figure 3A, bar groups 4–9). These results indicated that ISKNV-vSOCS expression inhibited the IFN-α-stimulated activities of both ISRE promoters.

Dose-dependent assays were performed to further confirm whether the activation of ISRE promoters by IFN-α was inhibited by ISKNV-vSOCS. Briefly, cells were transfected with increasing amounts of ISKNV-vSOCSmyc (0–200 ng) and stimulated with IFN-α (5000 U) for 8 h. The RLA levels were then detected. The results show that significantly lower RLA levels in cells transfected with higher amounts of ISKNV-vSOCSmyc (Figure 3B). RLA levels in cells transfected with 1 and 5 ng of ISKNV-vSOCSmyc were inhibited by 60% to 80%, indicating that ISKNV-vSOCS inhibited the activities of ISRE promoters stimulated by IFN-α in a dose-dependent manner.

### ISKNV-vSOCS Interacts with Jak1 Protein and Inhibits its Tyrosine Kinase Activity

**Figure 4:**
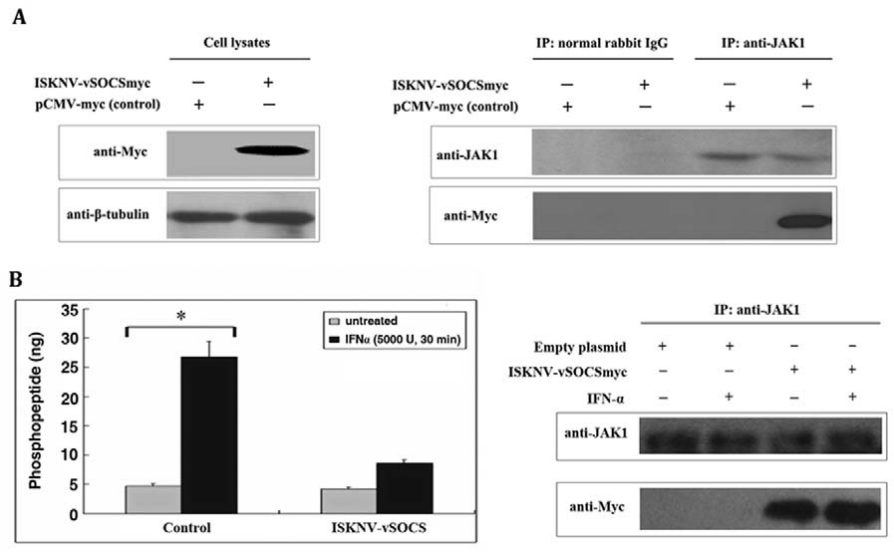
vSOCS interacted with Jak1 protein and inhibited the Jak1 tyrosine kinase activity *in vitro*. (A) Immunoprecipitation (IP) assay. Cells were transfected with pCMV-myc (empty plasmid) as negative control, or with ISKNV-vSOCS. Myc-tagged protein and tubulin were detected by anti-Myc and anti-β-tubulin antibodies in cell lysates (left plane) after 36 h of transfection. The cell lysates were immunoprecipitated with normal rabbit IgG or anti-Jak1 antibody, and then detected by western blotting using anti-Jak1 or anti-myc antibody (right plane). (B) Direct inhibition of Jak kinase activity by ISKNV-vSOCS *in vitro.* Cells were transfected with empty vector (control) or ISKNV-vSOCSmyc plasmid, and Jak1 was immunoprecipitated with anti-Jak1 antibody. A part of the immunoprecipitation complex was used to incubate with RTK substrate at room temperature for 1 h (left plane). The rest were detected by Western blotting using anti-Jak1 or anti-Myc antibody. Phosphorylated substrates were detected at an absorbance of 450 nm with a reference wavelength of 655 nm after adding mouse anti-PY20-HRP, TMB solution, and Stop Solution. The gray columns represent the cells without IFN-α stimulation, whereas the black columns represent the cells with IFN-α (5000 U) stimulation for 30 min. One out of six representative experiments is shown. Error bars represent the mean ± S.D. (n = 6). *P<0.05.

An immunoprecipitation assay was performed using cell lysates at 36 h post-transfection to investigate the mechanisms by which ISKNV-vSOCS inhibits the Jak/Stat signaling pathway. Jak1 precipitation with anti-Jak1 antibody and Jak1 was detected by western blotting to confirm the presence of Jak1 in both the control cells (transfected with the empty plasmid) and the cells expressing Myc-tagged ISKNV-vSOCS. Figure 4A (left plane) shows that Jak1 was obviously present in both the control cells and cells expressing ISKNV-vSOCS. Immunoprecipitation was subsequently performed (Figure 4A, right plane). No Myc-tagged ISKNV-vSOCS was detected in the precipitated proteins when the control cell lysate was precipitated with anti-Jak1 antibody because no ISKNV-vSOCS was expressed. Myc-tagged ISKNV-vSOCS was detected by the anti-Myc antibody when the recombinant lysate from cells expressing Myc-tagged ISKNV-vSOCS was precipitated with anti-Myc antibody, indicating that ISKNV-vSOCS was expressed. No Myc-tagged ISKNV-vSOCS was detected in the precipitated proteins when the recombinant lysate was precipitated with normal rabbit IgG. However, Myc-tagged ISKNV-vSOCS was detected by the anti-Myc antibody when the cell lysate was precipitated with anti-Jak1 antibody. In summary, these results suggest that recombinant ISKNV-vSOCS interacted with Jak1.

The tyrosine kinase activity of Jak family proteins is triggered by IFN [30]. An ELISA-based non-radioactive protein tyrosine kinase (PTK) activity assay (Chemicon, USA) was performed in vitro *t*o test Jak1 tyrosine kinase activity. Jak1 in both the control and recombinant cell lysates was subjected to immunoprecipitation with anti-Jak1 antibody and was used for the in vitro assays. The resulting protein complexes were detected by anti-Jak1 and anti-Myc antibodies (Figure 4B, right plane). The ability of Jak1 tyrosine kinase to phosphorylate PTK substrate was reflected by phosphopeptide production. Figure 4B (left plane) shows that, without IFN-α stimulation, Jak1 from both the control and recombinant cell lysates (containing Myc-tagged ISKNV-vSOCS) produced low levels of phosphopeptide (4.7 ng and 4.2 ng, respectively). However, the phosphopeptide produced by Jak1 from the control cell lysate after IFN-α stimulation significantly increased (∼5.8 fold, 26.8 ng), whereas the phosphopeptide produced by Jak1 from the recombinant cell lysate after IFN-α stimulation increased only by ∼1-fold (8.5 ng) compared with the cell lysate with no IFN-α stimulation. These results suggest that the expression of recombinant ISKNV-vSOCS protein impaired the tyrosine kinase activity of endogenous Jak1 protein.

### ISKNV-vSOCS Inhibits IFN-α-activated Phosphorylation of Stat1 and Sta3

**Figure 5:**
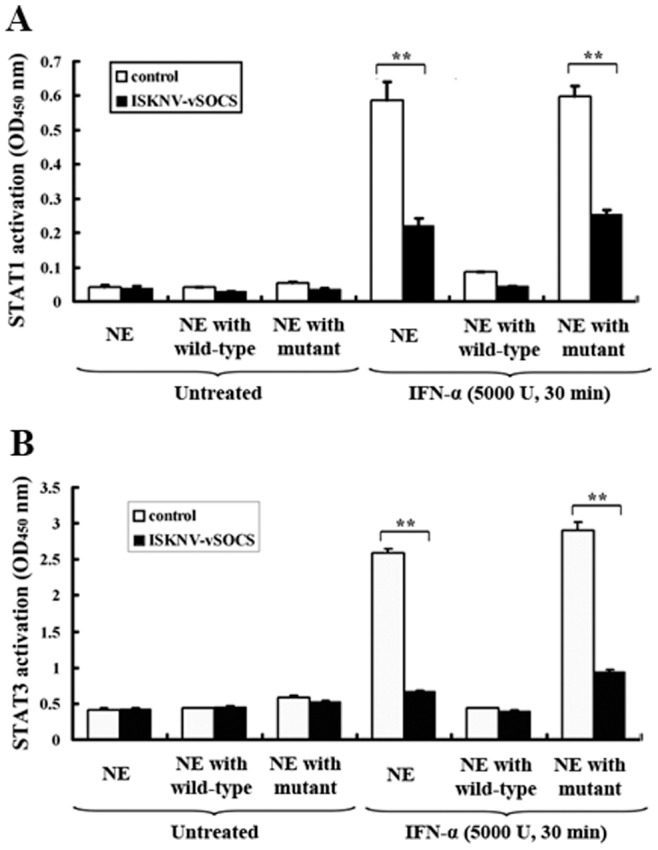
ISKNV-vSOCS inhibited the phosphorylation of Stat1 (A) and Stat3 (B) protein. Cells were treated with IFN-α (5000 U) for 30 min, and nuclear extracts were prepared and quantified at 24 h after transfection. The level of phosphorylated Stat1/3 protein was detected by absorbance at 450 nm according to the instructions of the manufacturer. The white and black columns represent the control and the ISKNV-vSOCS-containing nuclear extracts, respectively. Nuclear extracts were incubated with immobilized oligonucleotide containing the Stat consensus binding site (5′-TTC CCG GAA-3′) in a 96-well plate without competitor oligonucleotide (bar groups 1 and 4), and with wild-type oligonucleotide (bar groups 2 and 5) or mutant oligonucleotides (bar groups 3 and 6). Error bars represent the mean ± S.D. (n = 3). **P<0.01.

Phospho-STAT1 (p-STAT1) and phospho-STAT3 (p-STAT3) were detected using the TransAM™ Stat family transcription factor assay to determine whether the activation of Stat transcription factors was also inhibited by ISKNV-vSOCS [31]. Briefly, cells were stimulated with or without IFN-α (5000 U) for 30 min, and nuclear extracts were extracted and incubated with Stat-specific oligonucleotide to detect the activities of p-STAT1α and p-STAT3. The activities of STAT1α and STAT3 transcription factors were indicated by the absorbance at 450 nm (Figure 5). The results show that the activities of STAT1α (Figure 5A) and STAT3 (Figure 5B) in cells without IFN-α stimulation were low regardless of ISKNV-vSOCS expression (Figure 5, bar groups 1–3). However, the activities of STAT1α and STAT3 in the cells with IFN-α stimulation were significantly higher in the control cells (∼10- and 5-fold for STAT1α and STAT3, respectively) compared with the cells expressing recombinant ISKNV-vSOCS (∼5- and 1.5-fold STAT1α and STAT3, respectively) (Figure 5, bar group 4). The high number of activities of STAT1α and STAT3 in the control cells were inhibited by the wild-type oligonucleotide (>90%) (Figure 5B, bar group 5), but not by the mutant oligonucleotide (Figure 5, bar group 6). These results suggest that the expression of ISKNV-vSOCS inhibited the transcription activities of STAT1α and STAT3.

### ISKNV-vSOCS Point Mutants Suppresses the Inhibition of Jak/Stat Signaling

**Figure 6:**
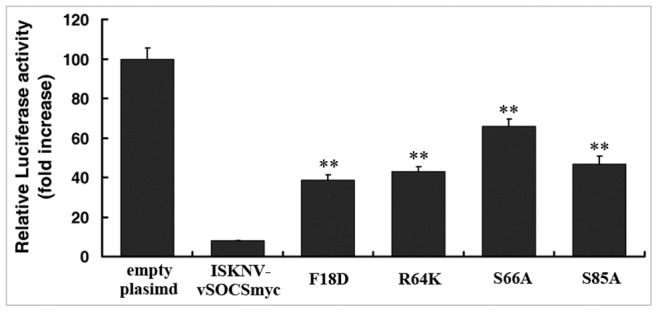
The point-mutations of ISKNV-vSOCS altered its inhibitory activity in the ISRE-promoter luciferase reporter genes. Cells were transfected with ISKNV-vSOCSmyc or its mutants, and then treated with IFN-α (5000 U) for 8 h. The activity of the ISRE-promoter luciferase reporter genes was detected. Relative luciferase activity in the cells transfected with empty plasmid after IFN treatment was arbitrarily set as 100. Error bars represent the mean ± S.D. (n = 3). **P<0.01.

Limnander et al. [32] demonstrated that the phosophorylation of nontyrosine residues in SOCS1 protein disrupts the degradation of Jak kinases. Several point mutations in ISKNV-vSOCS were constructed and the activities of mutant proteins were analyzed using dual-luciferase assays to further investigate the functions of ISKNV-vSOCS. Our results (Figure 6) show that IFN-activated Jak/Stat signaling was significantly inhibited by wild-type ISKNV-vSOCS (by comparing bars 2 and 1). However, mutations in the KIR (F18D) and SH2 domains (R64K, S66A, and S85A) of ISKNV-vSOCS significantly suppressed the inhibitory activities of mutant proteins on Jak/Stat signaling activation (Figure 6). Mutation agents in the interactions between ISKNV-vSOCS and Jak1 were also observed by Co-IP assay (Figure S1). F18D and R64K mutations in ISKNV-vSOCS correspond to F59D and R105K mutations in mouse SOCS1, where SOCS1 mutants impeded the activity of SOCS1 [13]. Mutations of two functional serine residues (serine-66 and serine-85) in ISKNV-vSOCS also strongly affected its inhibitory activities. These results have not been reported in other SOCS proteins**.**

### ISKNV-ΔvSOCS Virus Induces the Jak/Stat Signal Pathway in MFF-1 Cells

**Figure 7:**
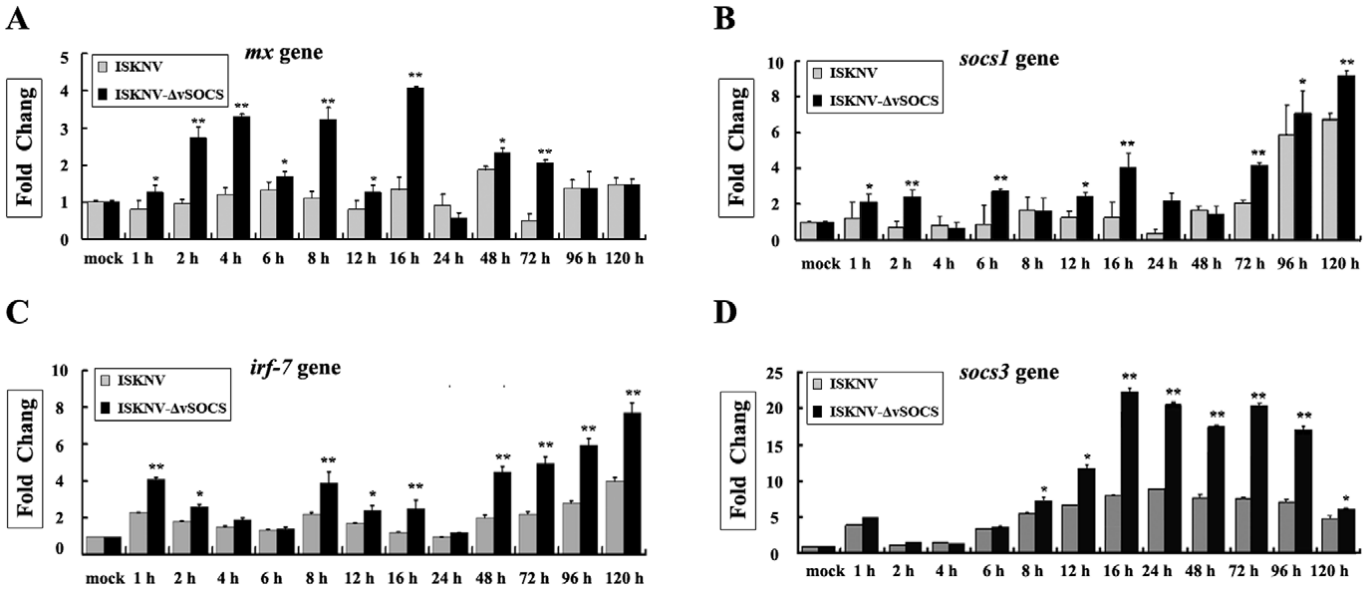
Real-time RT-PCR analysis of *mx* (A), *socs1* (B), *irf7* (C), and *socs3* (D) genes expressed the MFF-1 cells in response to ISKNV and ISKNV-ΔvSOCS viruses. Expression of β-actin from the same RNA samples was used as control. The expression levels of *mx*, *irf7*, *socs1,* or *socs3* gene at 0 h was arbitrarily set as 1. The grey and black columns indicate the gene expressions in cells infected with wild-type ISKNV and ISKNV-ΔvSOCS viruses, respectively. All data were analyzed by Q-gene statistics add-in, followed by unpaired sample t-test. Error bars represent the mean ± S.D. (n = 3). *P<0.05, **P<0.01.

*Mx*, *irf7*, *socs1*, and *socs3* genes, which are the downstream effector genes of IFN-induced Jak/Stat signaling, were strongly activated in mandarin fish cells after the previously described poly(I:C) stimulation [33], [34]. A mutant ISKNV that lacked the *vsocs* gene (ISKNV-ΔvSOCS) was constructed using homologous recombination, where the *vsocs* gene was replaced with green fluorescent protein (GFP) in the ISKNV-ΔvSOCS genome (unpublished data). Time-course expressions of *mx* and *socs1* genes were detected after the cells were infected with wild-type ISKNV and ISKNV-ΔvSOCS using quantitative real-time PCR to assess the function of ISKNV-vSOCS in ISKNV-infected cells. The results show that expressions of *mx* gene remained low in the period of 1–24 h and slightly increased in the period of 48–120 h after infection with wild-type ISKNV. However, the expression of the *mx* gene significantly increased at 1 h, peaked at 16 h (about 4-folds), and remained at a relatively high level within 48–120 h after infection with ISKNV-ΔvSOCS virus compared with wild-type ISKNV virus (Figure 7A). Similarly, expressions of *socs1* (Figure 7B), *irf7* (Figure 7C), and *socs3* (Figure 7D) genes were higher in cells infected with ISKNV-ΔvSOCS virus than with those infected with wild-type ISKNV virus. These results suggest that IFN signaling can be triggered by ISKNV virus deficiency in *vsocs* gene.

## Discussion

A vSOCS from the virus, with functions similar to vertebrate SOCS1 protein, was identified for the first time in this study. Over-expressed ISKNV-vSOCS in HepG2 cells interacted with Jak1 protein to inhibit its tyrosine kinase activity, and impaired the phosphorylation and transcription activity of Stat1 and Stat3 proteins. Moreover, the expressions of *mx*, *irf7*, *socs1,* and *socs3* genes were induced in the MFF-1 cells infected by the mutant virus (ISKNV-ΔvSOCS), but not by the wild-type ISKNV virus, suggesting that vSOCS serves as a suppressor that inhibits IFN-induced Jak/Stat signal transduction pathway in infected cells.

vSOCS was not only present in ISKNV, but was also found in other viral genomes of the genus *Megalocytivirus* in the family *iridoviridae*. We previously reported that the OSGIV ORF 99R encoded a putative SOCS [29]. Eaton et al. reannotated and defined the core set of iridovirus genes using comparative genomic assay to analyze the family *iridoviridae* [35]. They classified a new putative ORF 95.5R (from nucleotide 91477 to 91935 in the genome) in the RBIV genome, which was highly similar to ISKNV ORF103R (vSOCS) [28]. Moreover, an undefined ORF (from nucleotide 91537 to 91926 in the genome), which was an analogue of ISKNV-vSOCS in the LYCIV genome, was identified in this study [27]. Therefore, all four sequenced genomes in the genus *Megalocytivirus* contain ORF-encoding vSOCS proteins, including ISKNV ORF103R, OSGIV ORF 99R, RBIV ORF 95.5R, and the LYCIV undefined ORF. However, vSOCS only existed in the megalocytivirus and may be used as a typical gene to distinguish megalocytiviruses from other viruses.

Interestingly, megalocytivirus vSOCS shares a similar architecture with SOCS1, but lacks a SOCS-box domain (Figure 1). Therefore, megalocytivirus vSOCS was classified as a new SOCS family. The SOCS box of the SOCS family proteins can interact with the Elongin B/C complex, which acted as an E3 ubiquitin protein ligase to recruit the ubiquitin proteasome system [36]. SOCS1 promoted the ubiquitination and proteasomal degradation of TEL-Jak2 fusion protein in a SOCS box-dependent fashion [17]. The SOCS box does not only function in the degradation of associated target signal molecules, but is also involved in regulating SOCS protein levels [37]. The interaction between SOCS1 and Elongin B/C complex presumably contributed to the degradation of SOCS1 [38]–[40]. Recent studies demonstrated that SOCS2 bound to the SOCS box of all SOCS family, which accelerated the turnover of SOCS proteins [41]. The levels of SOCS1, as a potent signaling inhibitor, must be tightly regulated. The degradation mechanism of SOCS1 remains vague, but the SOCS box requirement of SOCS protein degradation was clearly demonstrated. vSOCS does not possess a SOCS box, thus, they may have longer half-life and prolonged inhibitory activity on IFNs-induced Jak/Stat signaling for megalocytivirus.

The mammalian SOCS1 directly bound to the Jaks through its SH2 domain, and inhibited the Jaks kinase activity via the KIR domain [42]. F18D and R64K vSOCS mutants were demonstrated based on F59D and R105K SOCS1 mouse mutants to impair the function of vSOCS. In addition, phosphorylation of nontyrosine residues in SOCS1 disrupted the interaction of SOCS1 with Elongin B/C complex, which led to the inhibited degradation of activated Jak proteins [32]. The role of potential phosphorylation sites in vSOCS on the inhibition function was not confirmed. Thus, several serine/threonine/tyrosine residue mutations in ISKNV-vSOCS, which were then mutated to alanine, were constructed. Results showed that mutations in the SH2 domain (S66A and S85A) of ISKNV-vSOCS altered the inhibitory activity of vSOCS on IFN-α induced Jak/Stat signaling (Figure 6), whereas the mutations of other serine residues were not affected (Figure S2). These results suggested that serine-66 and serine-85 in the SH2 domain of ISKNV-vSOCS are important in the interaction between vSOCS and Jak1 protein.

The IFNs-induced Jak/Stat signaling cascade responded to the viral infection. Several viruses developed various strategies to inhibit the IFNs-induced Jak/Stat signal transduction pathway to escape the antiviral immunity of the host [43]. The capability of several members of the *paramyxoviridae* family to block the Jak/Stat signaling pathway led to the impaired biological activity of IFNs. The hepatitis C virus encoded a suppressor that decreased the phosphorylation of Stat tyrosine and strongly inhibited the IFN-α-induced signal transduction [44]. The measles virus encoded a non-structural V protein that inhibited the phosphorylation of tyrosine residues on Stat1, Stat2, and Jak1 proteins in Hela cells after IFN-β stimulation [45]. The adenovirus E1A protein inhibited the formation of ISGF3 transcriptional complex by competing Stat1/2 protein binding with CREB-binding protein/p300 and blocked the IFN-α/β and IFN-γ signal transduction pathway [46]–[48]. Simian virus 5 facilitated the ubiquitination and subsequent proteasome-mediated degradation of Stat1 [49]. Several members of the *flaviviruses* family have also been confirmed to inhibit IFN signaling. The Japanese encephalitis virus blocked the phosphorylation of tyrosine residues on TYK2, Stat1, Stat2, and Stat3 [50]. The dengue virus encoded a protein that interacted with and retained the phosphorylated Stat1 in the cytoplasm and also reduced the Stat2 expression, leading to IFN signaling inhibition [51], [52]. The West Nile virus prevented the phosphorylation and activation of Jak1 and Tyk2 [53], [54]. In addition, other RNA viruses have also been reported to inhibit the Jak/Stat signaling pathway, including rabies virus, Ebola virus, and vesicular stomatitis virus [55]–[57]. HPV-18, a member of the *Papillomaviridae* family of dsDNA viruses, expressed an E6 oncoprotein that interacted with Tyk2, thereby impairing Jak/Stat activation [58]. The current study was first to report the ability of a vSOCS protein of megalocytiviruses in inhibiting the IFN-α-induced Stat1/3 signaling pathway through interaction with Jak1 protein. In addition, the IFN-γ-mediated reporter gene activities were also inhibited by ISKNV-vSOCS (Figure S3), whereas this inhibition was not observed in the ISKNV mutants of F18D, R64K, S66A, and S85A (Figure S4). Our results suggested that megalocytiviruses may have developed a novel strategy different from other viruses to escape the interferon-induced antiviral mechanisms via vSOCS proteins. ISKNV-vSOCS interacted with Jak1 protein and inhibited the IFN-induced antiviral immunity. This process may prolong the inhibitory activity because of the lacking SOCS-box domain, which is involved in the recruitment of the ubiquitin proteasome system.

A phylogenetic tree was constructed with the vSOCS proteins and other SOCS family proteins from the vertebrate to further study the evolutionary origin of vSOCS. vSOCS from the genus *Megalocytivirus* was more closely related to the SOCS1 proteins of fishes than that from frogs, chickens, platypuses, and mammals. The megalocytivirus possibly gained its *vsocs* genes from fish during evolution. Compared with the SOCS1 of fishes, the vSOCS lacks a SOCS box, which may be attributed to the following two reasons. First, the vSOCS genes of the genus *Megalocytivirus* may have originated from a “full-length” SOCS1 gene and lost its SOCS box during evolution. Second, the genus *Megalocytivirus* may have selectively obtained a SOCS1 gene from a host without a SOCS1 box.

In conclusion, we demonstrated that the vSOCS of the megalocytivirus suppressed Stat1/3 signaling, which led to impaired IFN-α response. The discovery of vSOCS and further research on its multiple functions will help identify antiviral targets and develop more efficient antiviral drugs.

## Materials and Methods

### Cells and Antibodies

HepG2 (ATCC HB8065) cells were cultured in a complete Dulbecco’s modified Eagle medium (DMEM, Gibco, USA) supplemented with 10% FBS (Gibco, USA) and maintained at 37°C. Mandarin fish fry (MFF-1) cells were cultured in DMEM supplemented with 10% FBS at 27°C under humidified atmosphere containing 5% CO_2_, as described previously [34].

Anti-Jak1 antibody was obtained from Upstate Biotechnology (Lake Placid, NY, USA), and anti-Myc tag antibody was purchased from Clontech (Tokyo, Japan). Anti-phospho-Stat1 (Tyr701) and anti-phospho-Stat3 (Tyr705) were purchased from Cell Signaling Technology (Beverly, MA, USA). Anti-β-tubulin antibody was purchased from Epitomics, Inc. (Burlingame, CA, USA).

### Plasmid Construction and Mutations

Genomic DNA and cDNA were extracted from the spleens of ISKNV-infected mandarin fish. Based on the DNA sequence of ISKNV ORF103R, a pair of primers was designed as follows: 5′-GGA ATT CGG TGC TCC CCG ACG TCA TCG-3′ (sense), which contains an EcoRI site, and 5′-CCG CTC GAG TTA CTG TGT TTG CAC CCG CTT C-3′ (antisense), which contains an XhoI site. DNA fragment was amplified by PCR, digested with EcoRI and XhoI (Takara Shuzo, Kyoto, Japan), and then inserted into the pCMV-myc (Clontech, Tokyo, Japan) vector. The resultant plasmids were named ISKNV-vSOCSmyc. The point-mutations of KIR (F18D) and SH2 domains (R64K, S66A, and S85A) were prepared with ISKNV-vSOCSmyc as template according to the site-directed gene mutagenesis kit (Beyotime Biotechnology, Jiangsu, China). All plasmids were confirmed by sequencing.

### Sequence Analysis

The amino acid sequence of ISKNV-vSOCS was analyzed using the Simple Modular Architecture Research Tool (SMART) (http://smart.embl-heidelberg.de/) and basic local alignment tool (BLAST) (http://www.ncbi.nlm.nih.gov/BLAST). Sequence alignments were performed using ClustalX v1.83, and edited using the GeneDoc v2.60 software. The phylogenetic tree was constructed using the Bootstrap N-J method of the Molecular Evolutionary Genetics Analysis (MEGA) software version 4.0 [59]. Bootstrap sampling was reiterated 1,000 times. All data were retrieved from the GenBank or the Ensemble database. The accession numbers were also indicated.

### Luciferase Reporter Assay

The pISRE-luc reporter plasmid (Clontech, Tokyo, Japan) was designed to monitor the induction of the interferon-α-triggered signaling transduction pathway [60], [61]. A luciferase vector without *cis*-acting element that responds to a particular pathway was used as a negative control (pTA-luc). Cells were cultured at 5×10^4^ cells per well in a 48-well plate and co-transfected with 0.2 µg of pISRE-luc reporter plasmid, 20 ng of pRL-TK reporter plasmid, and 0.2 µg of pCMV-myc/ISKNV-vSOCSmyc plasmids (1 ng to 200 ng plasmids were employed in the dose-dependent assays) using Lipofectamine™ 2000 reagent (Invitrogen, USA). Cells were treated with recombinant interferon-α (IFN-α, PeproTech, USA) for 8 h at 24 h after transfection. Cell lysates were harvested and assayed for the luciferase activity using the dual-luciferase® reporter assay system (Promega, USA).

### Immunoprecipitation

Cells from 75 cm^2^ flasks were lysed using a modified RIPA lysis buffer (20 mM Tris, pH 7.5, 150 mM NaCl, 1% Triton X-100, 0.1 mM EDTA supplemented with 2 µg/mL pepstatin A, 5 µg/mL leupeptin, 5 µg/mL aprotinin, 1 mM PMSF, and 1 mM Na_3_VO_4_) containing protease inhibitor cocktail after 36 h of transfection. Pelleted cellular debris was collected by centrifugation. Supernatants were precleared with protein A/G plus agarose for 1 h at 4°C on a rocker platform. A 5 µL of anti-Jak1 antibody was added to the supernatants and incubated at 4°C on a rocker platform overnight. The mixture was immunoprecipitated by incubating with fresh protein A/G plus agarose beads for 2 h. The beads were then washed four times with RIPA lysis buffer and once with PBS. The bound proteins were eluted from the beads by boiling with 1×SDS sample loading buffer. Anti-myc antiserum was used as primary antibody to detect immunoprecipitation.

### Tyrosine Kinase Activity Assay

Jak1 proteins were immunoprecipitated by the A/G plus agarose beads as described above. The activities of Jak1 kinase were assessed using the protein tyrosine kinase (PTK) activity assay kit (Chemicon, California, USA) according to the protocol of the manufacturer. Briefly, the PTK reactions were mixed with the beads in 10 µL of 5×tyrosine kinase assay buffer (300 mM HEPES, 25 mM MgCl_2_, 25 mM MnCl_2_, 15 µM Na_3_VO_4_, 25 mM ATP, 12.5 mM dithiothreitol), 10 µL of protein tyrosine kinase substrate (10 µg/mL), 20 µL of ultrapure water, and 10 µL of 5×ATP/MgCl_2_ solution (5 mM ATP, 50 mM MgCl_2_ in TBS, pH7.2). The mixture was then incubated for 60 min at 37°C. The enzyme reactions were stopped with 120 mM EDTA, and then the 50 µL products were transferred to streptavidin-coated 96-well strip and subsequently incubated at 37°C for 30 min. The phosphorylated substrates were detected at an absorbance of 450 nm after incubation with mouse anti-PY20-HRP, TMB substrate, and Stop Solution.

### Western Blotting

Jak1 and β-tubulin proteins, anti-myc, anti-Jak1, and anti-β-tubulin were employed, respectively, to detect Myc-tagged ISKNV-vSOCS. Immunosignals were detected by enhanced chemiluminescence reagent (Pierce, USA) and exposed to polyvinylidene diﬂuoride membranes by Hyperfilm (Amersham Biosciences, Piscataway, NJ).

### Stat Family Transcription Factor Assay

TransAM™ Stat family transcription factor assay (Active Motif, Carlsbad, USA) is an ELISA-based assay used to detect and quantify the transcription factor activation for Stat signaling pathways studies [58]. The nuclear proteins were extracted from HepG2 cells using the nuclear extraction kit (Active Motif) according to the protocol of the manufacturer. Stat1/3 activations were analyzed based on the directions of the TransAM™ kit. Briefly, equal quantities of nuclear proteins were incubated with specific oligonucleotide for 1 h. The oligonucleotide contained the Stat consensus binding site: 5′-TTC CCG GAA-3′ and immobilized in a 96-well plates. Free wild-type consensus oligonucleotide (containing the Stat consensus binding site) was used as competitor for the Stat binding to monitor the specificity of the assay. The mutation consensus oligonucleotide was used as negative control. Approximately 100 µL of diluted anti-phospho-Stat1/3 antibody was added to each well and incubated for 1 h. The wells were washed, and 100 µl of diluted HRP-conjugated antibody was added to each well and incubated for 1 h without agitation. The wells were washed again four times, followed by incubation with 100 µl of RT developing solution for 10 min. The reaction was terminated by adding 100 µl of stop solution. Absorbance was measured at 450 nm, with a reference wavelength of 655 nm.

### Virus Purifications and Infections

The viruses were propagated in MFF-1 cells, and the infected cells were then lysed and subjected to three freeze-thawed cycles. Cell debris were pelleted at 10,000 rpm for 30 min at 4°C. The supernatants were centrifuged at 30,000 rpm in a Beckman-type 70 Ti rotor for 60 min at 4°C. The virus pellets were then resuspended and layered onto 30%, 40%, 50%, and 60% (w/v) sucrose gradients. The viral band was formed between 40% and 50% sucrose layer after centrifugation at 100,000 g for 2 h in a Beckman SW41 Ti rotor. The viral band was subsequently collected. The virions were resuspended and centrifuged at 100,000 g at 4°C for 30 min. The virion pellets were then finally collected and stored at −80°C.

Equal multiplicity of infections (MOI = 10) of wild-type ISKNV and ISKNV-ΔvSOCS viruses were prepared for immune challenged experiments. MFF-1 cells were cultured in 6-well plates at 2×10^6^ cells per well overnight. Each cell was then infected with wild-type ISKNV and ISKNV-ΔvSOCS viruses, respectively. The cells were harvested for RNA preparation using Trizol reagents (Invitrogen, USA) at various times (2, 4, 6, 8, 16, 24, 48, 72, 96, and 120 h) post-infection. All RNAs were reverse transcribed to cDNAs as described previously [34].

### Quantitative Real-time PCR

The expressions of *Mx*, *irf7*, *socs1,* and *socs3* genes were performed by real-time quantitative PCR on a LightCycler (Roche Diagnostics, Switzerland) using SYBR premix Ex Taq™ (Takara, Japan), as described previously [33], [34]. The expression levels of each transcript were normalized to β-actin expression, which was used as an internal housekeeping control. All data were analyzed using Q-gene statistics add-in followed by unpaired sample *t*-test [62].

### Statistical Analysis

All data were expressed as a mean ± standard deviation (SD). Statistical significance was accepted at p<0.05, whereas high significance was accepted at p<0.01.

## Supporting Information

Figure S1 **The point-mutations of ISKNV-vSOCS interacted with Jak1 protein via immunoprecipitation assay.** Cells were transfected with ISKNV-vSOCSmyc or its mutants. Cell lysates were immunoprecipitated with anti-Jak1 antibody, and then detected by Western blotting using anti-myc antibody at 36 h after transfection.
(TIF)

Figure S2 **The point-mutations in ISKNV-vSOCS altered its inhibitory activity on ISRE-promoter luciferase reporter genes.** Cells were transfected with ISKNV-vSOCSmyc or its mutants, and then treated with IFN-α (5000 U) for 8 h. The activities of the ISRE-promoter luciferase reporter genes were detected. Relative luciferase activity in the cells transfected with empty plasmid after IFN-α treatment was arbitrarily set as 100. Error bars represent the mean ± S.D. (n = 3). **P<0.01
(TIF)

Figure S3 **Activities of the gamma-interferon-activation sites (GAS)-promoter luciferase reporter gene.** (A) IFN-γ-responsive GAS-luc promoter activity. Cells were treated with recombinant IFN-γ (1∼100 ng/mL) for 8 h at 24 h after transfection. The gray columns represent the RLA levels in cells transfected with empty plasmid, whereas the black columns represent the RLA levels in cells transfected with ISKNV-vSOCSmyc plasmid. RLA level in cells transfected with TA-luc reporter gene instead of ISRE-luc reporter gene was used as negative control. RLA levels of cells transfected with empty plasmid without stimulation were arbitrarily set as 1. (B) Activities of reporter genes in cells transfected with increasing amounts of ISKNV-vSOCSmyc plasmid. Cells were transfected with different amounts of ISKNV-vSOCSmyc plasmid (1∼200 ng), treated with IFN-γ (50 ng/mL) for 8 h, and then GAS-luc activity was analyzed. RLA levels in cells transfected with empty plasmid after IFN-γ treatment were arbitrarily set as 1. Error bars represent the mean ± S.D. (n = 3). **P<0.01
(TIF)

Figure S4 **The point-mutations in ISKNV-vSOCS altered its inhibitory activity on GAS-promoter luciferase reporter genes.** Cells were transfected with ISKNV-vSOCSmyc or its mutants and then treated with IFN-γ (50 ng/mL) for 8 h. The activities of the GAS-promoter luciferase reporter gene were detected. Relative luciferase activity in cells transfected with empty plasmid after IFN-γtreatment was arbitrarily set as 100. Error bars represent the mean ± S.D. (n = 3). **P<0.01
(TIF)
