# Supplementary figures and images for: A Novel Viral SOCS from Infectious Spleen and Kidney Necrosis Virus: Interacts with Jak1 and Inhibits IFN-α Induced Stat1/3 Activation
Source: PLoS One. 2012 Jul 23;7(7):e41092. doi: 10.1371/journal.pone.0041092 (PMC3402483; doi:10.1371/journal.pone.0041092)

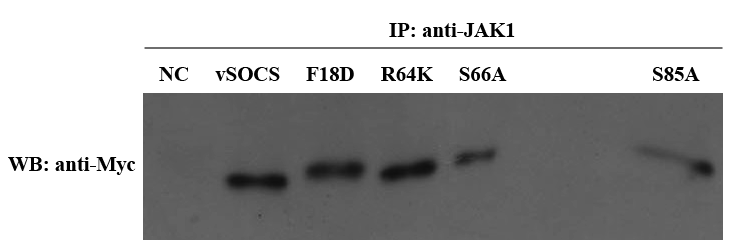

Supplement: Figure S1 — The point-mutations of ISKNV-vSOCS interacted with Jak1 protein via immunoprecipitation assay. Cells were transfected with ISKNV-vSOCSmyc or its mutants. Cell lysates were immunoprecipitated with anti-Jak1 antibody, and then detected by Western blotting using anti-myc antibody at 36 h after transfection. (TIF) [file pone.0041092.s001.tif]

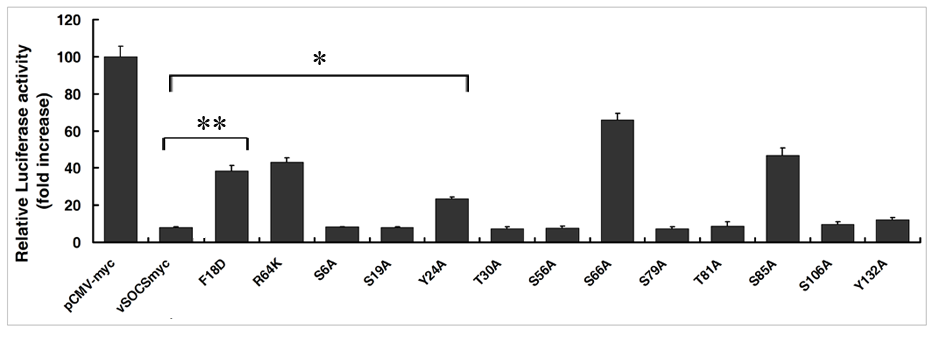

Supplement: Figure S2 — The point-mutations in ISKNV-vSOCS altered its inhibitory activity on ISRE-promoter luciferase reporter genes. Cells were transfected with ISKNV-vSOCSmyc or its mutants, and then treated with IFN-α (5000 U) for 8 h. The activities of the ISRE-promoter luciferase reporter genes were detected. Relative luciferase activity in the cells transfected with empty plasmid after IFN-α treatment was arbitrarily set as 100. Error bars represent the mean ± S.D. (n = 3). **P<0.01 (TIF) [file pone.0041092.s002.tif]

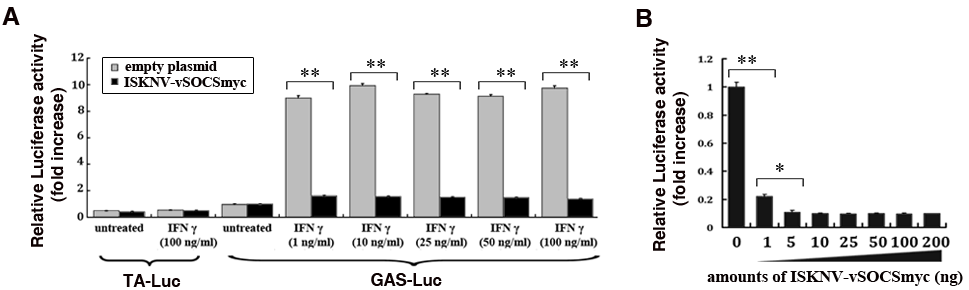

Supplement: Figure S3 — Activities of the gamma-interferon-activation sites (GAS)-promoter luciferase reporter gene. (A) IFN-γ-responsive GAS-luc promoter activity. Cells were treated with recombinant IFN-γ (1∼100 ng/mL) for 8 h at 24 h after transfection. The gray columns represent the RLA levels in cells transfected with empty plasmid, whereas the black columns represent the RLA levels in cells transfected with ISKNV-vSOCSmyc plasmid. RLA level in cells transfected with TA-luc reporter gene instead of ISRE-luc reporter gene was used as negative control. RLA levels of cells transfected with empty plasmid without stimulation were arbitrarily set as 1. (B) Activities of reporter genes in cells transfected with increasing amounts of ISKNV-vSOCSmyc plasmid. Cells were transfected with different amounts of ISKNV-vSOCSmyc plasmid (1∼200 ng), treated with IFN-γ (50 ng/mL) for 8 h, and then GAS-luc activity was analyzed. RLA levels in cells transfected with empty plasmid after IFN-γ treatment were arbitrarily set as 1. Error bars represent the mean ± S.D. (n = 3). **P<0.01 (TIF) [file pone.0041092.s003.tif]

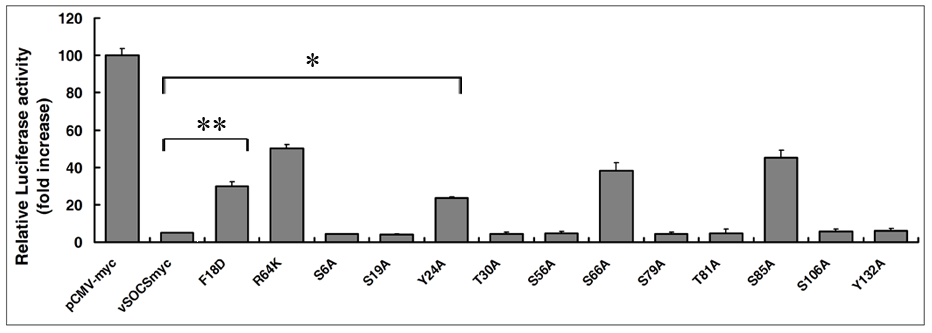

Supplement: Figure S4 — The point-mutations in ISKNV-vSOCS altered its inhibitory activity on GAS-promoter luciferase reporter genes. Cells were transfected with ISKNV-vSOCSmyc or its mutants and then treated with IFN-γ (50 ng/mL) for 8 h. The activities of the GAS-promoter luciferase reporter gene were detected. Relative luciferase activity in cells transfected with empty plasmid after IFN-γtreatment was arbitrarily set as 100. Error bars represent the mean ± S.D. (n = 3). **P<0.01 (TIF) [file pone.0041092.s004.tif]
